# Feasibility and Safety of Paclitaxel-Coated Balloon Angioplasty for the Treatment of Intracranial Symptomatic In-Stent Restenosis

**DOI:** 10.3389/fneur.2020.00774

**Published:** 2020-08-11

**Authors:** Haowen Xu, Xiaojie Fu, Yongjie Yuan, Tao Quan, Zibo Wang, Kaihao Han, Guo Liu, Sheng Guan

**Affiliations:** Department of Neurointerventional Radiology, First Affiliated Hospital of Zhengzhou University, Zhengzhou, China

**Keywords:** intracranial atherosclerotic stenosis, in-stent restenosis, drug-coated balloon, angioplasty, stroke

## Abstract

**Objective:** Symptomatic in-stent restenosis (sISR) is the major cause of medium- or long-term cerebral infarctions in patients who underwent percutaneous transluminal angioplasty and stenting for severe intracranial atherosclerotic stenosis. This study aims to evaluate the feasibility and safety of paclitaxel-coated balloon (PCB) angioplasty for the treatment of intracranial sISR.

**Methods:** We report 11 cases of PCB angioplasty for intracranial sISR. Lesion locations and number were as follows: intracranial internal carotid artery (*n* = 4), M1 segment of middle cerebral artery (MCA) (*n* = 1), V4 segment of vertebral artery (*n* = 6). The technical success rate, periprocedural complications, and short-term outcome were retrospectively analyzed.

**Results:** All procedures were successfully performed without periprocedural complication. Asymptomatic vessel dissection after PCB inflation occurred in one case. Postprocedural diffusion-weighted imaging (DWI) showed new asymptomatic ipsilateral infarction in one case. All 11 cases did not experience ipsilateral stroke or death within 30 days or ischemic stroke in the territory of the target artery between 31 and 90 days after procedure.

**Conclusion:** This preliminary study indicates that PCB angioplasty is feasible and safe for the treatment of intracranial sISR. Further studies are needed to clarify its efficiency and long-term outcome.

## Introduction

Intracranial atherosclerotic stenosis (ICAS) responsible for 33–37% of acute ischemic strokes in Asian populations (1). WASID (Warfarin-Aspirin Symptomatic Intracranial Disease) trial demonstrated that more than 20% of medical-treated symptomatic ICAS patients had poor outcomes, driving rapid development in endovascular treatment (2). Percutaneous transluminal angioplasty and stenting (PTAS) has been evolving as a potential treatment for ICAS patients with recurrent stroke despite medical treatment. However, the use of PTAS in ICAS became increasingly debated since the publish of SAMMPRIS (Stenting and Aggressive Medical Management for the Prevention of Recurrent Stroke Intracranial Stenosis) trial (3) and VISSIT (Vitesse Intracranial Stent Study for the Ischemic Therapy) trial (4). Both trials indicated that symptomatic ICAS patients treated with stenting had significantly higher periprocedural morbidity and mortality than that treated with aggressive medical management (AMM).

However, SAMMPRIS trial still demonstrated that 12.2% of patients with symptomatic severe ICAS developed ipsilateral stroke or death within 1 year despite AMM treatment (3), suggesting significant need for alternative treatment strategies. In 2019, the WEAVE (Wingspan Stent System Post Market Surveillance) trial (5) reported that with precise patient selection following the on-label usage guidelines, a low periprocedural complication rate (2.4%) of Wingspan stenting for ICAS could be achieved by experienced interventionalists. This joyful result demonstrated that PTAS is a promising therapy for symptomatic ICAS patients who are refractory to AMM.

High in-stent restenosis (ISR) rate is one of the discouraging results of intracranial stenting. In the SAMMPRIS trial, during a median follow-up of 35 months, various degrees of ISR were found in 66.7% of patients with infarction and 80% of patients with transient ischemic attack (TIA) who received adequate vascular imaging examination (6). The 1-, 2-, and 3-years rates for symptomatic ISR (sISR) of patients treated with Wingspan stenting were 9.6, 11.3, and 14%, respectively (6). Symptomatic ISR is the major cause of medium- or long-term ipsilateral stroke after intracranial stenting. Bare balloon angioplasty and restenting are the two mostly reported interventional strategies to deal with sISR, but the restenosis rate is still high, and the efficiency remains unknown. The application of drug-coated balloons (DCBs, mostly paclitaxel-coated) angioplasty has been proven as a promising effective method to prevent and treat sISR in coronary and peripheral arteries in abundant studies (7–9). The use of DCB angioplasty for intracranial sISR was reported in few case reports. In this study, we evaluated the feasibility and safety of paclitaxel-coated balloon (PCB) angioplasty for the treatment of intracranial sISR.

## Methods

### Patient Selection

We conducted a retrospective review of ICAS patients treated with PTAS in our center (including Heyi, Zhengdong, and Huiji Branch Hospitals) from January 2018 to July 2019. Patients who developed sISR and treated with PCB angioplasty were retrospectively analyzed. Patients treated with PTAS and had any of the following events were identified (6): (1) ischemic stroke in the territory of the stenting artery, (2) cerebral infarction with transient signs in the territory, or (3) TIA was associated with the territory. In-stent restenosis was determined by digital subtraction angiography (DSA) and defined as >50% stenosis within or immediately adjacent (within 5 mm) of the implanted stent and >20% absolute luminal loss (6, 10). Symptomatic ISR was defined as probable or definite ISR-associated ischemic symptoms in the territory (6).

The criteria are as follows. Inclusion criteria were as follows: (1) 18–80 years old; (2) intracranial sISR; (3) baseline modified Rankin score <3; (4) patient understands the purpose and requirements of this therapy and has provided informed consent. Exclusion criteria were as follows: (1) intracranial or extracranial arterial dissection, moyamoya disease, vasculitis, radiation-induced vasculopathy, fibromuscular dysplasia; (2) a severe neurological deficit that renders the patient incapable of living independently; (3) dementia or psychiatric problem that prevents the reliable follow-up; and (4) comorbid conditions that may limit survival to <5 years.

A total of 151 ICAS patients who received successful intracranial stenting in our center (including Heyi, Zhengdong, and Huiji Branch Hospitals) were retrospectively reviewed; 85.4% (129/151) of them had valid angiographic follow-ups, and 15 patients (11.6% in 129) developed sISR. In the 15 patients with sISR, three of them rejected PCB angioplasty; one patient did not meet the criteria because of lung cancer, and 11 patients were finally included. All patients or their authorized family members were fully informed the benefits and risks of endovascular treatment and off-label use of the PCB. Clinical and imaging data of the subjects were retrospectively analyzed. This study was approved by the ethics committee of the First Affiliated Hospital of Zhengzhou University (approval no. 2019-KY-195). The privacy of patients was strictly protected.

### Procedure

The preprocedural management included physical examination, brain magnetic resonance (MR) imaging, or high-resolution MR imaging of the target artery, Mini Mental State Examination (MMSE) score before and after procedure, and dual antiplatelet therapy with 100 mg of aspirin and 75 mg of clopidogrel daily at least 5 days.

Paclitaxel-coated balloon angioplasty was performed under general anesthesia. Heparin was titrated during the procedure to maintain activated clotting time between 250 and 300 s. The ISR grade was assessed according to the WASID trial. In this study, we used SeQuent Please (B. Braun, Berlin, Germany) to treat intracranial sISR. The paclitaxel-loading dosage is 3 *u*g/mm^2^ and 16% of which will finally be implanted in the vessel wall. SeQuent Please is relatively rigid, which makes it challenging to use in patients with tortuous intracranial vasculature and requires rapid navigation and location to the target lesion; therefore, we used intracranial support catheter (5F or 6F Navien, ev3, Irvine, CA, USA) in most cases to support use of the PCB. Via the guiding catheter or Navien, the ISR lesion was crossed with a 0.014-inch Synchro microguidewire (Stryker Neurovascular, Salt Lake City, UT, USA) and predilated with bare balloon (Gateway balloon; Boston Scientific, Maple Grove, MN, USA). The bare balloon was then exchanged for a similarly sized PCB and centered across the lesion within 90 s. The DCB was then slowly inflated and kept at work pressure for 60 s. The ISR degree of residual stenosis was confirmed by DSA after the PCB withdrawn. The technical success of PCB angioplasty was defined as less 50% residual stenosis and stable antegrade perfusion (2b/3a) with no vessel dissection, perforation, or distal embolization (11). If the residual stenosis was more than 50% or there were vessel dissection, stent placement could be considered.

After the procedure, patients were typically monitored in neuro critical care units for 24 h. Postprocedurally, the systolic blood pressure was kept under 130 mm Hg. All patients who underwent PCB angioplasty were continued on 100 mg aspirin and 75 mg clopidogrel daily for 3 months and 100 mg of aspirin daily thereafter. Postprocedural MR imaging and MR angiography were performed within 2 weeks after treatment. The clinical follow-up was scheduled for 1, 3, 6, and 12 months and yearly thereafter.

### Data Collection and Analysis

The following data were collected: demographic characters of all patients such as age, sex, location of the target artery, date of last stent implantation, periprocedural complications of last stenting procedure, MMSE scores before and after the procedure, date and feature of the symptomatic neurological symptoms, Mori classification of ISR (12), degree of ISR, size of bare, and PCB used in the angioplasty, residual stenosis after dilation, and occurrence, type, and severity of all periprocedural complications.

The feasibility of PCB angioplasty for the treatment of intracranial ISR was determined by the following: (1) if the PCB can be safely transferred to the target lesion within 90 s despite significant tortuous access; (2) if the PCB can be safely inflated in target vessel for 60 s; (3) if the ISR grade was safely improved after PCB angioplasty. Safety of PCB angioplasty in intracranial ISR was determined by the following: (1) there was no hemorrhagic stroke due to microguidewire/microcatheter perforation or vessel rupture or hyperperfusion injury within 30 days after procedure; (2) there was no ischemic stroke due to distal embolization, perforator occlusion, and vessel dissection; (3) there was no stroke or death within 30 days after PCB angioplasty or ischemic stroke in the territory of the target artery between 31 and 90 days after procedure (3). Descriptive statistical methods were applied in this study.

## Results

### Patient Characteristics

Eleven patients underwent PCB angioplasty for intracranial sISR, and their data were retrospectively analyzed in this study; 90.1% (10/11) of the patients were male. Ages ranged from 40 to 71 years with a mean of 56.0 years. The prevalence of dyslipidemia, hypertension, and diabetes was 72.7% (8/11), 36.4% (4/11), and 36.4% (4/11); 45.5% (5/11) of the sISR located in anterior cerebral circulation. The baseline characteristics are presented in Table 1.

**Table 1:** Baseline demographic characteristics.

**Baseline demographic characteristics (*n* = 13)**	***n* (%)**
Male, *n* (%)	10 (90.1)
Age, mean ±*SD* (range), years	56.0 ± 10.2 (40–71)
Hyperlipidemia	8 (72.7)
Hypertension	4 (36.4)
Diabetes mellitus	4 (36.4)
Body mass index (18–24)	6 (54.5)
Smoker (former or current)	9 (81.8)
Moderate or vigorous exercise	4 (36.4)
Valid aggressive medical management	3 (27.3)

### Standard-Reaching Rate of AMM

All subjects received AMM after the first PTAS treatment. On the baseline of this study, 36.4% (4/11) of the subjects had low-density lipoprotein (LDL) level >1.8 mg/dL, 36.4% (4/11) had hypertension (with systolic/diastolic blood pressure >140/90 mm Hg), 18.2% (2/11) had poor blood glucose control (with blood glycated hemoglobin level >6.0%), 18.2% (2/11) were current smokers, and 36.4% (4/11) lack moderate or vigorous exercise. In total, 27.3% (3/11) of the subjects achieved the goal of AMM (Table 1).

### Technique and Clinical Outcome

**Figure 1:**
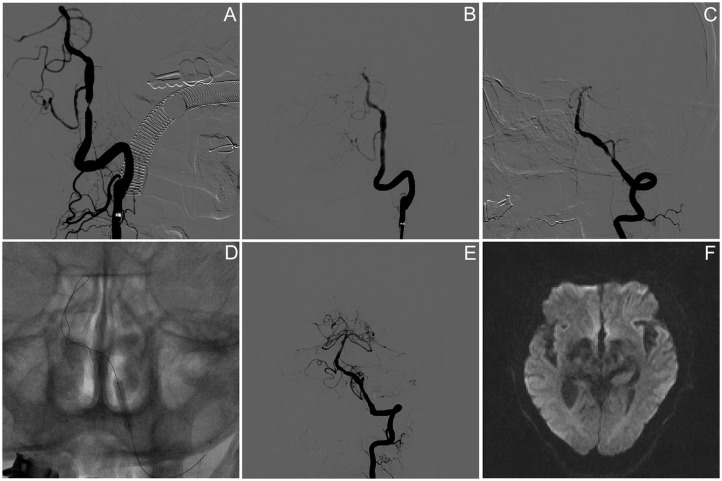
Paclitaxel-coated balloon angioplasty for intracranial sISR. **(A)** Symptomatic severe vertebral artery atherosclerotic stenosis despite of aggressive medical management. **(B)** Angiography results after PTAS using wingspan stent. **(C)** Symptomatic ISR because of the discontinuance of aggressive medical management. **(D)** Drug-coated balloon dilatation. **(E)** Angiographic result after the DCB angioplasty. **(F)** Brain DWI result on day 5 after procedure presented no new infarction.

In a total of 11 PCB angioplasty cases, all PCBs were transferred to the target lesion within 90 s and inflated for at least 60 s. Paclitaxel-coated balloon angioplasty was technically successful in 90.1% (10/11) of patients. Asymptomatic vessel dissection after PCB inflation occurred in one patient (9.1%). No distal embolization or snowplow effect was seen during the navigation, location, inflation, deflation, and withdrawal of the PCB catheter. The preprocedure stenosis was 76.4 ± 8.3%, and postprocedure stenosis was 19.5 ± 9.6% (Table 1); 9.1% (1/11) of the subjects had asymptomatic ipsilateral infarction based on MR imaging after procedure. There was no symptomatic stroke or death within 30 days after DCB angioplasty or ischemic stroke in the territory of the target artery between 31 and 90 days after procedure. The lesion and procedural characteristics of the 11 patients are presented in Table 2. The PCB angioplasty procedure in a patient with sISR was presented in Figure 1.

**Table 2:** Lesion and procedural characteristics.

**Lesion and procedural characteristics (*n* = 13)**	***n* (%)**
**Lesion location**	
Internal carotid artery	4 (36.4)
Middle carotid artery	1 (9.1)
Vertebral artery	6 (54.5)
**Stenosis degree, Mean** **±*****SD*****, %**	
Before procedure	76.4 ± 8.3
After DCB angioplasty	19.5 ± 9.6
Technique success	10 (90.1)
Symptomatic ipsilateral infarction	0 (0)
Asymptomatic ipsilateral infarction	1 (9.1)
Vessel dissection	1 (9.1)
Perforation	0 (0)
Distal embolization	0 (0)
Decreased MMSE score	0 (0)

## Discussion

In this single-center retrospective pilot study, we found that PCB angioplasty was feasible and safe in the treatment of patients with intracranial sISR.

High periprocedural complication rate and intracranial ISR are the two main factors that limit the use of PTAS in treatment of ICAS. As discussed before, the WEAVE trial demonstrated that with experienced interventionalists, precise patient selection, and on-label usage guidelines, PTAS has excellent safety profile and a low periprocedural complication rate (2.4%) in ICAS patients (5), suggesting the urgent need for intracranial ISR research.

The researches that focus on intracranial ISR are limited. The reported prevalence of intracranial ISR ranges from 14.4 to 30% (6, 13, 14). In the stenting arm of SAMMPRIS trial, of 183 patients without a periprocedural primary endpoint, intracranial ISR was found in 70.6% (24/34) of patients with symptomatic infarction or TIA during a median follow-up of 35.0 months. Symptomatic ISR occurred in at least one of seven patients by 3 years of follow-up and was likely responsible for the majority of non-procedural cerebral infarctions (6). In a prospective study of 226 Chinese ICAS patients treated with PTAS, during a median follow-up of 10.1 months, 25.2% (*n* = 57) patients developed intracranial ISR and 26.3% (15/57) of which were symptomatic (15). These studies indicate that intracranial ISR is a key risk factor that affects the long-term outcome of ICAS patients treated with PTAS.

The mechanism of intracranial ISR remains unknown. Unlike the ISR research in cardiac and peripheral vessels, the basic study of intracranial ISR is relatively few. Clinical prospective studies have demonstrated that age, diabetes mellitus, stent type, lesion location, and history of smoking are risk factors in the development of ISR after intracranial stenting, which are similar with cardiac and peripheral ISR (13, 16, 17). In this study, only three of the 11 patients (27.3%) achieved the goal of AMM since the last PTAS, indicating that patient compliance may also affect the progress of intracranial ISR. In the SAMMPRIS trial, even under the rigorous follow-up strategy, more than 30% patients cannot achieve target blood pressure and LDL level. The poor adherence and low goal-achieving rate of AMM are indeed concerning problems, indicating more efforts should be done in postdischarge treatment. Early elastic return, relocation of axially transmitted plaque, reorganization of thrombus, neointima formation, vascular remodeling, neoatherosclerosis, platelet aggregation resolution (18), and inflammation are the pathogenic mechanisms that underlie peripheral ISR (12). However, considering the difference between cerebral vascular and peripheral vascular, the intrinsic mechanism of intracranial ISR still needs further research.

The optimal management of patients with intracranial sISR remains unclear (6). Currently, the most reported methods include medical and interventional treatment. Although small sample studies showed that dual antiplatelet and stain therapy may be effective in intracranial sISR patients (6), the long-term efficiency of medical treatment is uncertain. Interventional treatment includes balloon angioplasty and restenting. Wu et al. (19) used balloon angioplasty and restenting to treat 21 patients with intracranial sISR; one patient experienced perforator stroke after procedure, and one patient had acute cerebral infarction during follow-up; 90.5% (19/21) patients had alleviated ISR grade and good outcome. Cardiac studies demonstrated that bare balloon angioplasty and restenting have a high restenosis rate in the treatment of ISR (12); the efficiency of its use for intracranial sISR needs further research (20).

Drug-coated balloon angioplasty has been officially recommended to treat coronary sISR. The balloon-carried drug, usually paclitaxel, can effectively inhibit smooth muscle cells proliferation and migration by irreversibly stabilizing intracellular microtubules (12). Some studies have reported the intracranial use of DCB angioplasty. Vajda et al. (21) reported that predilatation with SeQuent Please PCB followed by the deployment of Enterprise stent could significantly decrease the intracranial ISR rate to 3% in ICAS patients during average 8.9 months' follow-up. Predilatation using a conventional percutaneous transluminal coronary angioplasty (PTCA) balloon (Ryujin Plus Terumo) was performed in 13 cases (24%). The DCB angioplasty was attempted in 51 cases, and 23.6% failed (12 cases). The authors claimed the failure to difficult anatomy combined with the shaft thickness and the rigidity of the DCB-tip (21). Gruber et al. (22) compared the safety and efficacy between Neuro Elutax SV PCB angioplasty and routine PTAS in the treatment of symptomatic ICAS; they found both safety and efficacy were similar (complication rate: 0 vs. 18%, *P* = 0.21; technical success: 63 vs. 64%, *P* = 0.0.96, in DCB and PTAS groups, respectively). The DCB failure occurred in one case because of the difficult local anatomical conditions (22). In 2011, Zsolt Vajda et al. (20) first reported the use of DCB angioplasty for neurovascular ISR. They found the recurrent stenosis rate (9%) of DCB angioplasty arm was significantly lower than that of bare balloon angioplasty (50%). In four of 47 cases (8%), the DCB could not be navigated through the in-stent stenotic lesion, and the treatment of these lesions was finally performed with a conventional balloon (20). This encouraging result showed that DCB was a promising technique to treat intracranial sISR, but since then, few studies have further reported this technical development.

In this study, we retrospectively analyzed the feasibility and safety of PCB angioplasty in patients with intracranial sISR. We used PCB catheter, which has been reported for the intracranial use (11, 20, 21). Difficult local anatomical conditions combined with the rigidity of the DCB-tip are the most reported reasons that lead to the failure of PCB angioplasty in intracranial vascular (20–22). In our study, we found that with the help of intracranial support catheter and proper patient selection, PCB can be safely navigated to the target artery, even the distal portion of M1segment of middle cerebral artery. The technical success was achieved in 90.1% (10/11) of patients. One patient had asymptomatic vessel dissection after PCB inflation, which may be related to the stiffness of PCB catheter. This patient was treated with dual antiplatelet and stain therapy and had no symptoms during the 3-months follow-up. One patient had asymptomatic ipsilateral infarction after the procedure, which may be related to the microembolus during the angioplasty. No patients had decreased MMSE on day 5 after the procedure. There was no symptomatic stroke or death within 30 days or ischemic stroke in the territory of the target artery between 31 and 90 days after procedure.

This study has some important limitations. This descriptive study has a small sample size and lack of further follow-up. Based on the results of this study, we are enrolling more subjects and will continue the follow-up to investigate the long-term outcome of PCB angioplasty in intracranial sISR. This study showed the feasibility and safety of PCB angioplasty in patients with intracranial sISR. Further studies are needed to clarify its efficiency and long-term outcome.

## Data Availability Statement

The datasets generated for this study are available on request to the corresponding author.

## Ethics Statement

The studies involving human participants were reviewed and approved by Ethics Committee of the First Affiliated Hospital of Zhengzhou University (Approval No. 2019-KY-195). The patients/participants provided their written informed consent to participate in this study.

## Author Contributions

HX and XF wrote this manuscript. SG approved the final submission. All authors participated the surgery operations in 11 patients.

## Conflict of Interest

The authors declare that the research was conducted in the absence of any commercial or financial relationships that could be construed as a potential conflict of interest.

## References

[1] Arenillas JF. Intracranial atherosclerosis: current concepts. Stroke. (2011) 42:S20–3. 10.1161/STROKEAHA.110.597278 21164126

[2] Chimowitz MI Kokkinos J Strong J Brown MB Levine SR Silliman S. The warfarin-aspirin symptomatic intracranial disease study. Neurology. (1995) 45:1488–93. 10.1212/WNL.45.8.1488 7644046

[3] Chimowitz MI Lynn MJ Derdeyn CP Turan TN Fiorella D Lane BF. Stenting versus aggressive medical therapy for intracranial arterial stenosis. N Engl J Med. (2011) 365:993–1003. 10.1056/NEJMoa1105335 21899409 PMC3552515

[4] Zaidat OO Fitzsimmons B-F Woodward BK Wang Z Killer-Oberpfalzer M Wakhloo A. Effect of a balloon-expandable intracranial stent vs medical therapy on risk of stroke in patients with symptomatic intracranial stenosis. JAMA. (2015) 313:1240. 10.1001/jama.2015.1693 25803346

[5] Alexander MJ Zauner A Chaloupka JC Baxter B Callison RC Gupta R WEAVE trial. Stroke. (2019) 50:889–94. 10.1161/STROKEAHA.118.023996 31125298

[6] Derdeyn CP Fiorella D Lynn MJ Turan TN Cotsonis GA Lane BF. Nonprocedural symptomatic infarction and in-stent restenosis after intracranial angioplasty and stenting in the SAMMPRIS Trial (Stenting and Aggressive Medical Management for the Prevention of Recurrent Stroke in Intracranial Stenosis). Stroke. (2017) 48:1501–6. 10.1161/STROKEAHA.116.014537 28455321 PMC8204379

[7] Cassese S Ndrepepa G Kufner S Byrne RA Giacoppo D Ott I. Drug-coated balloon angioplasty for in-stent restenosis of femoropopliteal arteries: a meta-analysis. EuroIntervention. (2017) 13:483–9. 10.4244/EIJ-D-16-00735 28169215

[8] Cassese S Wolf F Ingwersen M Kinstner CM Fusaro M Ndrepepa G. Drug-coated balloon angioplasty for femoropopliteal in-stent restenosis. Circulation. (2018) 11:e007055. 10.1161/CIRCINTERVENTIONS.118.007055 30562083

[9] Kokkinidis DG Hossain P Jawaid O Alvandi B Foley TR Singh GD. Laser atherectomy combined with drug-coated balloon angioplasty is associated with improved 1-year outcomes for treatment of femoropopliteal in-stent restenosis. J Endovasc Therapy. (2018) 25:81–8. 10.1177/1526602817745668 29219030

[10] Gruber P Braun C Kahles T Hlavica M Anon J Diepers M. Percutaneous transluminal angioplasty using the novel drug-coated balloon catheter SeQuent Please NEO for the treatment of symptomatic intracranial severe stenosis: feasibility and safety study. J NeuroIntervent Surg. (2019) 11:719–22. 10.1136/neurintsurg-2018-014378 30415229

[11] Han J Zhang J Zhang X Zhang J Song Y Zhao W. Drug-coated balloons for the treatment of symptomatic intracranial atherosclerosis: initial experience and follow-up outcome. J NeuroInterventional Surg. (2019) 11:569–73. 10.1136/neurintsurg-2018-014237 30337378

[12] Buccheri D Piraino D Andolina G Cortese B. Understanding and managing in-stent restenosis: a review of clinical data, from pathogenesis to treatment. J Thoracic Dis. (2016) 8:E1150–62. 10.21037/jtd.2016.10.93 27867580 PMC5107494

[13] Investigators SS Stenting of Symptomatic Atherosclerotic Lesions in the Vertebral or Intracranial Arteries (SSYLVIA): study results. Stroke. (2004) 35:1388–92. 10.1161/01.STR.0000128708.86762.d6 15105508

[14] Groschel K Schnaudigel S Pilgram SM Wasser K Kastrup A. A systematic review on outcome after stenting for intracranial atherosclerosis. Stroke. (2009) 40:e340–7. 10.1161/STROKEAHA.108.532713 19182081

[15] Jin M Fu X Wei Y Du B Xu XT Jiang WJ. Higher risk of recurrent ischemic events in patients with intracranial in-stent restenosis. Stroke. (2013) 44:2990–4. 10.1161/STROKEAHA.113.001824 23963335

[16] Wabnitz A Chimowitz M. Angioplasty, stenting and other potential treatments of atherosclerotic stenosis of the intracranial arteries: past, present and future. J Stroke. (2017) 19:271–6. 10.5853/jos.2017.01837 29037013 PMC5647644

[17] Xiong Y Zhou Z Lin H Lin M Liu J Niu G. The safety and long-term outcomes of angioplasty and stenting in symptomatic intracranial atherosclerotic stenosis. Int J Cardiol. (2015) 179:23–4. 10.1016/j.ijcard.2014.10.081 25464398

[18] Mazighi M Maurice JPS Bresson D Szatmary Z Houdart E. Platelet aggregation in intracranial stents may mimic in-stent restenosis. Am J Neuroradiol. (2010) 31:496–7. 10.3174/ajnr.A1778 19833804 PMC7963984

[19] Wu ZH Qiu HC Hu SS Liu AF Wang K Zhou J. Interventional treatment of symptomatic intracranial in-stent restenosis. Zhonghua yi xue za zhi. (2018) 98:3017–20. 10.3760/cma.j.issn.0376-2491.2018.37.014 30392260

[20] Vajda Z Güthe TM Perez A Heuschmid A Schmid E Bäzner H. Neurovascular in-stent stenoses: treatment with conventional and drug-eluting balloons. Am J Neuroradiol. (2011) 32:1942–7. 10.3174/ajnr.A2644 21885715 PMC7966004

[21] Vajda Z Güthe T Perez MA Kurre W Schmid E Bäzner H. Prevention of intracranial in-stent restenoses: predilatation with a drug eluting balloon, followed by the deployment of a self-expanding stent. CardioVascular Interventional Radiol. (2013) 36:346–52. 10.1007/s00270-012-0450-9 22869043 PMC3595472

[22] Gruber P Garcia-Esperon C Berberat J Kahles T Hlavica M Anon J. Neuro Elutax SV drug-eluting balloon versus Wingspan stent system in symptomatic intracranial high-grade stenosis: a single-center experience. J NeuroInterventional Surg. (2018) 10:e32. 10.1136/neurintsurg-2017-013699 29627786

